# Supplementary material for: MIM-CyCIF: masked imaging modeling for enhancing cyclic immunofluorescence (CyCIF) with panel reduction and imputation
Source: Commun Biol. 2024 Apr 3;7:409. doi: 10.1038/s42003-024-06110-y (PMC10991424; doi:10.1038/s42003-024-06110-y)
Supplement: Supplementary file 2 — Supplementary information [file 42003_2024_6110_MOESM2_ESM.pdf]

## Supplementary Information

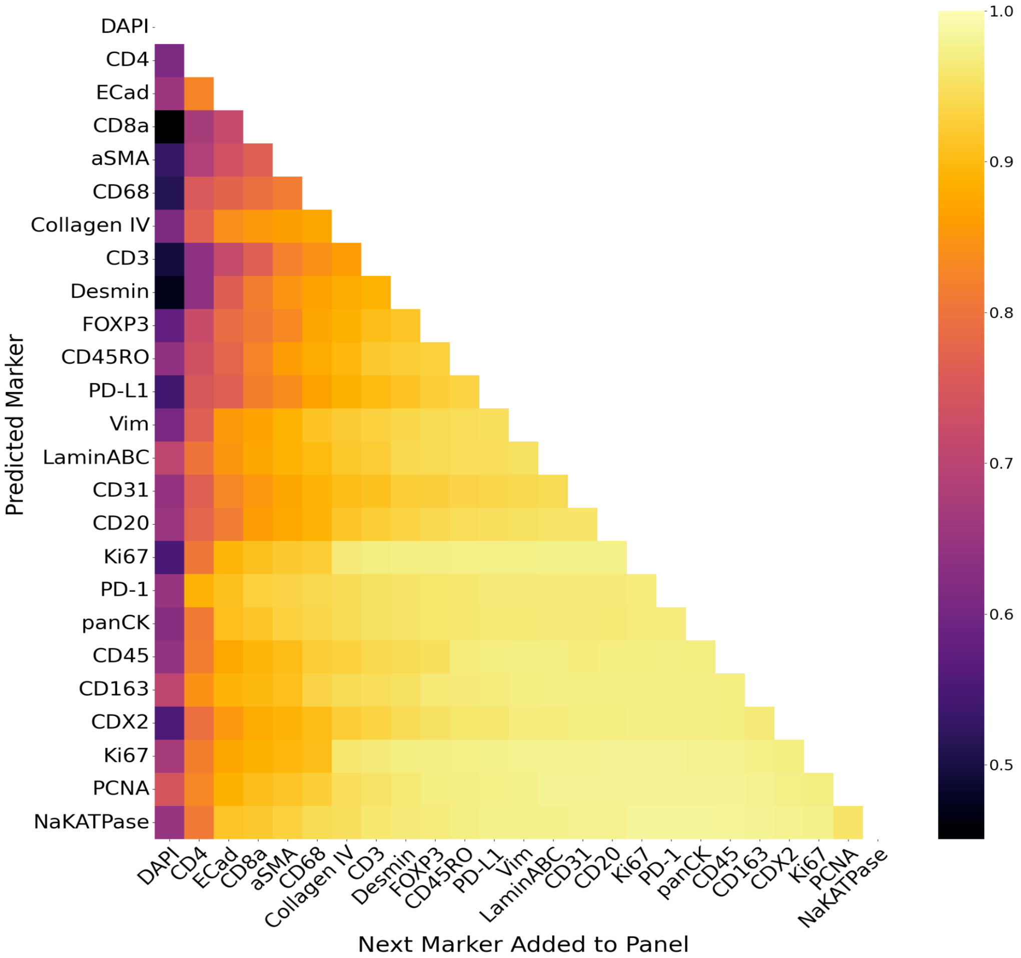


**Supplementary Figure 1| CRC Panel selection and marker prediction.** The ordering of markers in the CRC panel, determined by iterative selection, and the correlation for each predicted marker. A heatmap illustrates the mean Spearman correlation between actual and predicted mean intensities.

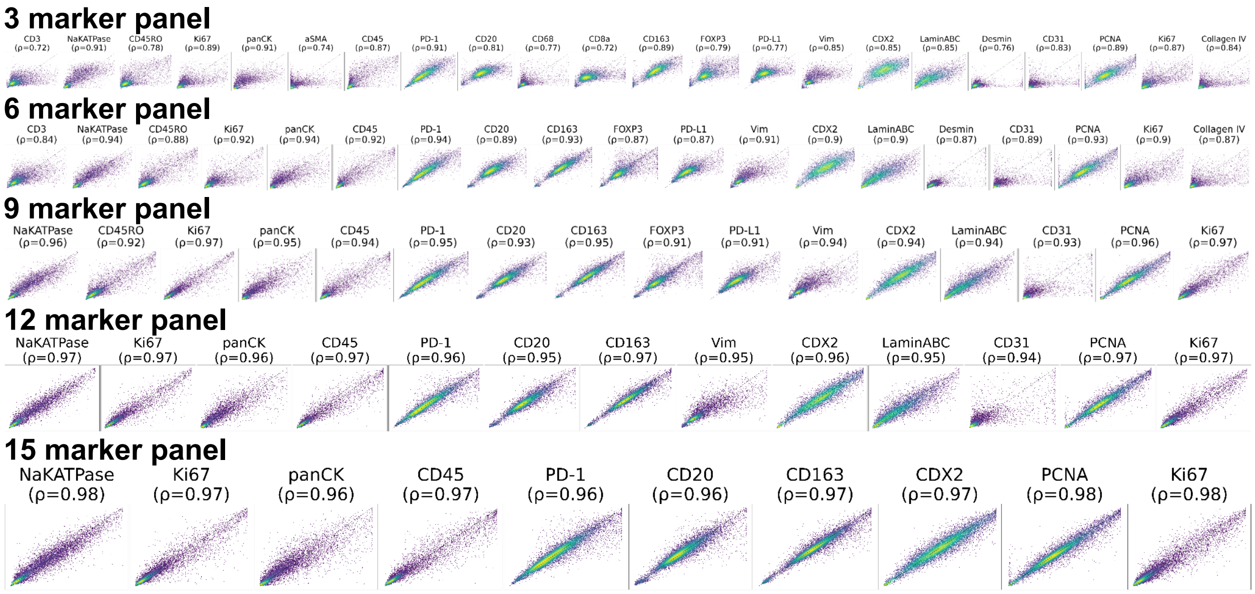


**Supplementary Figure 2| CRC marker prediction.** Real versus predicted single-cell mean intensity values. Plots of actual versus predicted single-cell mean intensity values are presented for reduced panel sizes of 3,6,9,12, and 15 markers, respectively. A random subset of 10,000 cells is shown. The Spearman correlation for each marker is indicated.

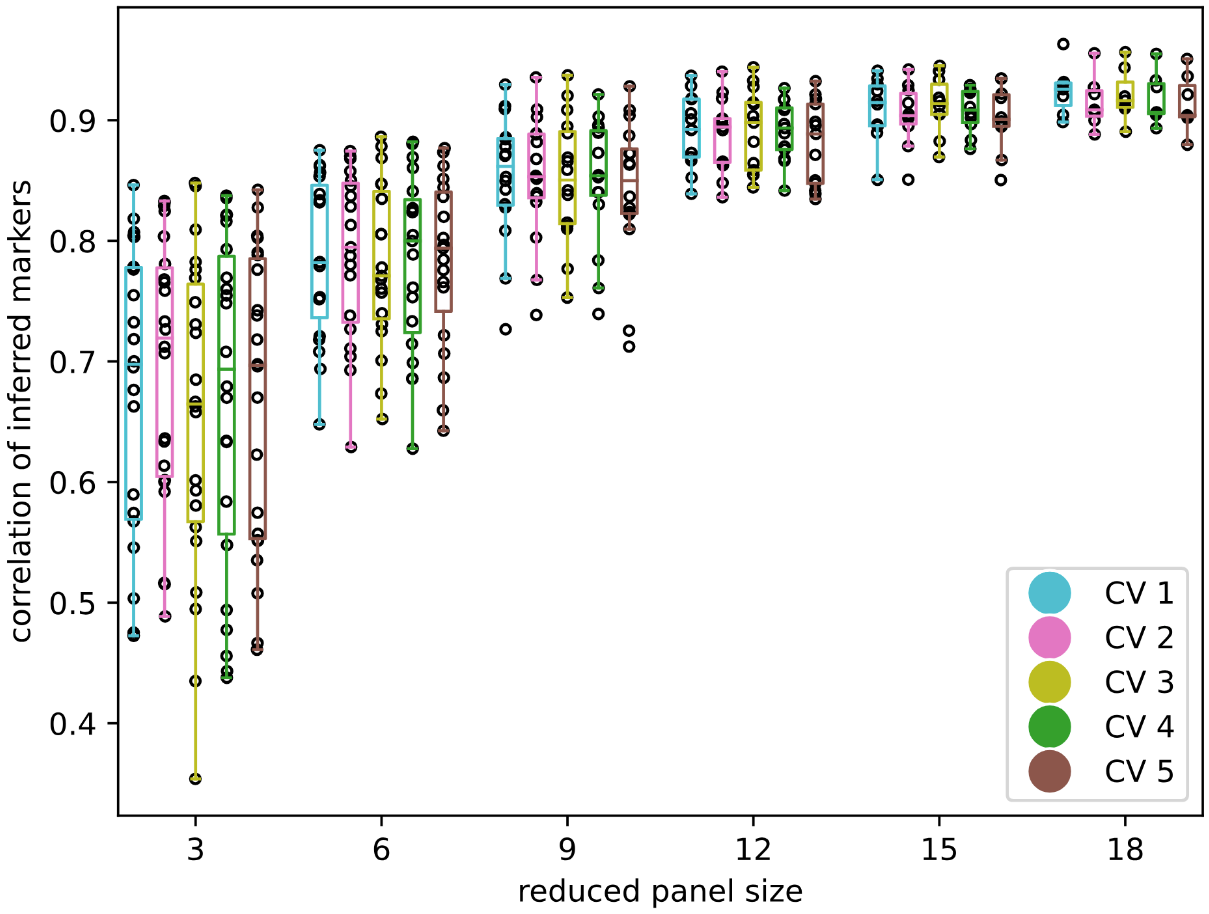


**Supplementary Figure 3| Cross-validation results for CRC TMA analysis across six distinct reduced panel sizes.** Cross-validation results, employing a 5-fold approach on the CRC TMA for six different panel sizes (3,6,9,12,15, and 18 markers), highlight the robustness and generalizability of the panel reduction over various withheld datasets.

**A**

**B**

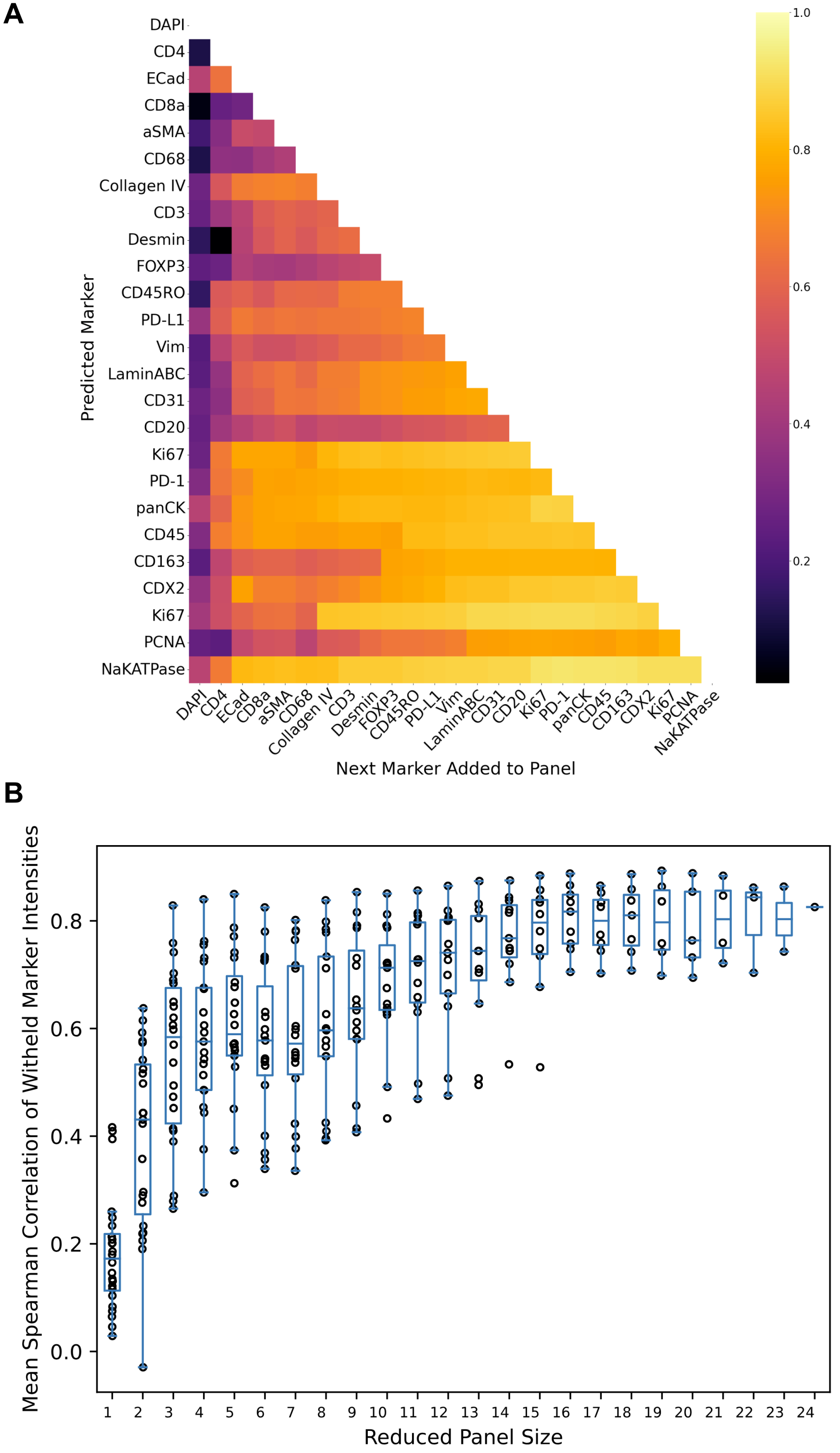


**Supplementary Fig. 4| Prediction Result for WSI Test Set. A.** Correlations for predicted markers on the CRC WSI dataset using reduced panels selected from the CRC TMA dataset. A heatmap illustrates the mean Spearman correlation between actual and predicted mean intensities. **B.** Performance of the pre-trained model on different reduced panel sizes.

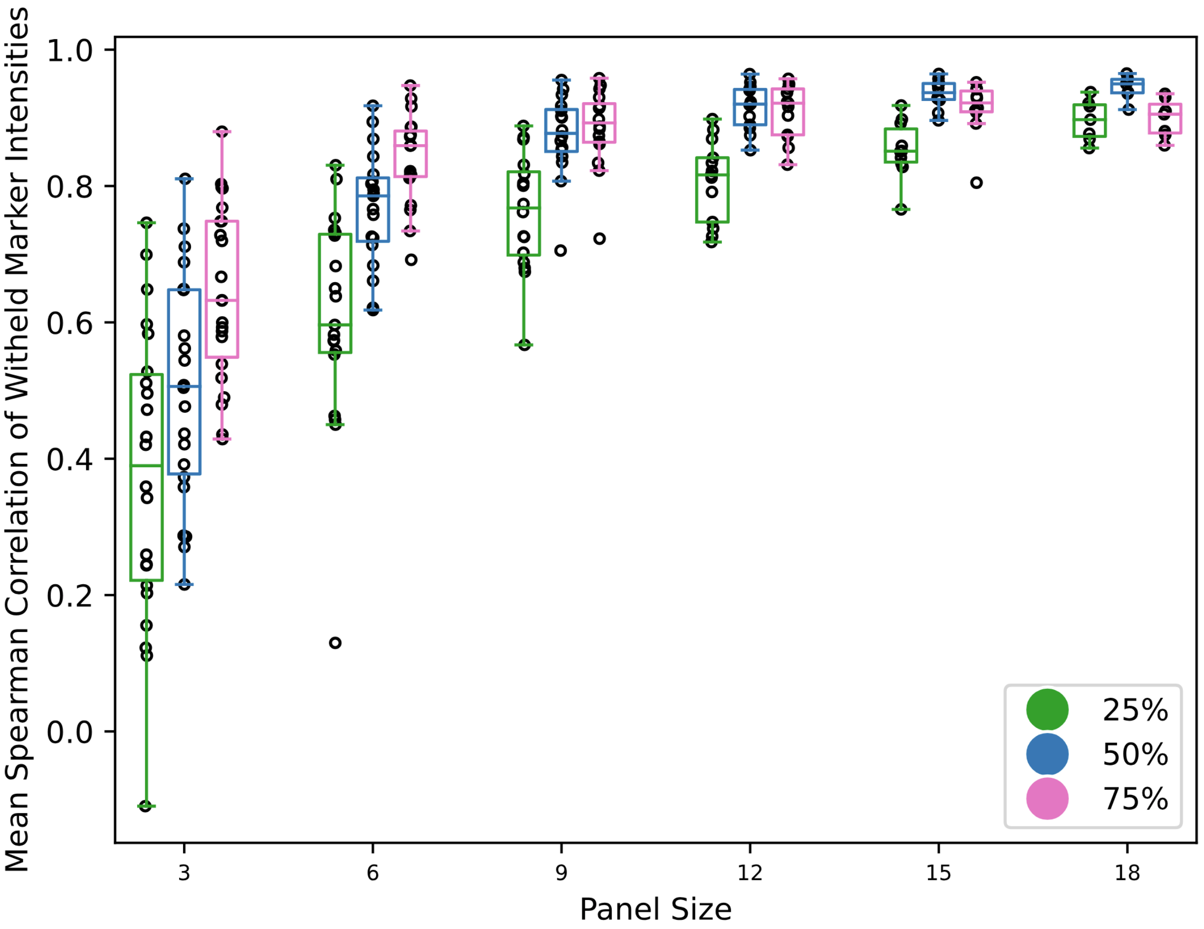


**Supplementary Fig. 5| Masking Ratio Evaluation in BC TMA.** The ratio of masked channels is fixed during training. Models trained with different ratios are compared by measuring the performance of different masking ratios in inference. The model trained at a 50% masking ratio performs the best across different masking ratios in inference.

**Supplementary Table. 1| CyCIF Biomarker Panels**

| | Breast Cancer CyCIF Panel | | | | Colorectal Cancer CyCIF Panel | | | |
|---|---|---|---|---|---|---|---|---|
| Cycle 1 | DAPI | CD3 | ERK-1 | hRAD51 | DAPI | CD3 | NaKATPase | CD45RO |
| Cycle 2 | DAPI | CyclinD1 | Vim | aSMA | DAPI | Ki67 | PanCK | aSMA |
| Cycle 3 | DAPI | ECad | ER | PR | DAPI | CD4 | CD45 | PD-1 |
| Cycle 4 | DAPI | EGFR | Rb | HER2 | DAPI | CD20 | CD68 | CD8a |
| Cycle 5 | DAPI | Ki67 | CD45 | p21 | DAPI | CD163 | FOXP3 | PD-L1 |
| Cycle 6 | DAPI | CK14 | CK19 | CK17 | DAPI | ECad | Vim | CDX2 |
| Cycle 7 | DAPI | LaminABC | AR | Histone H2AX | DAPI | LaminABC | Desmin | CD31 |
| Cycle 8 | DAPI | PCNA | PanCK | CD31 | DAPI | PCNA | Ki67 | Collagen IV |
