# Supplementary material for: MIM-CyCIF: masked imaging modeling for enhancing cyclic immunofluorescence (CyCIF) with panel reduction and imputation
Source: Commun Biol. 2024 Apr 3;7:409. doi: 10.1038/s42003-024-06110-y (PMC10991424; doi:10.1038/s42003-024-06110-y)
Supplement: Supplementary file 3 — Reporting Summary [file 42003_2024_6110_MOESM3_ESM.pdf]

nature portfolio

Corresponding author(s): Young Hwan Chang

Last updated by author(s): 02/23/2024

Reporting Summary

Nature Portfolio wishes to improve the reproducibility of the work that we publish. This form provides structure for consistency and transparency in reporting. For further information on Nature Portfolio policies, see our Editorial Policies and the Editorial Policy Checklist.

Statistics

For all statistical analyses, confirm that the following items are present in the figure legend, table legend, main text, or Methods section.

n/a	Confirmed	
☐	☒	The exact sample size (*n*) for each experimental group/condition, given as a discrete number and unit of measurement
☐	☒	A statement on whether measurements were taken from distinct samples or whether the same sample was measured repeatedly
☒	☐	The statistical test(s) used AND whether they are one- or two-sided<br>*Only common tests should be described solely by name; describe more complex techniques in the Methods section.*
☒	☐	A description of all covariates tested
☒	☐	A description of any assumptions or corrections, such as tests of normality and adjustment for multiple comparisons
☐	☒	A full description of the statistical parameters including central tendency (e.g. means) or other basic estimates (e.g. regression coefficient) AND variation (e.g. standard deviation) or associated estimates of uncertainty (e.g. confidence intervals)
☒	☐	For null hypothesis testing, the test statistic (e.g. *F*, *t*, *r*) with confidence intervals, effect sizes, degrees of freedom and *P* value noted<br>*Give P values as exact values whenever suitable.*
☒	☐	For Bayesian analysis, information on the choice of priors and Markov chain Monte Carlo settings
☒	☐	For hierarchical and complex designs, identification of the appropriate level for tests and full reporting of outcomes
☐	☒	Estimates of effect sizes (e.g. Cohen's *d*, Pearson's *r*), indicating how they were calculated

Our web collection on statistics for biologists contains articles on many of the points above.

Software and code

Policy information about availability of computer code

Data collection	As part of this paper all images at full resolution, all derived image data (e.g. segmentation masks), and all cell count tables will be publicly released via the NCI-recognized repository for Human Tumor Atlas Network (HTAN; https://humantumoratlas.org/) at Sage Synapse (associated Identifiers: HTAN TNP – TMA, OHSU_TMA1_004-XX) where XX represents TMA core ID. For CRC TMA, see source publication24 (DOI:10.1016/j.cell.2022.12.028).<br>All the source data for graphs and figures are available at the following link31: DOI:10.5281/zenodo.10724928.
Data analysis	All software used in this manuscript is detailed in the article's Methods section and its Supplementary Information. The associated scripts are freely available via GitHub as described at https://github.com/zacsims/IF_panel_reduction.

For manuscripts utilizing custom algorithms or software that are central to the research but not yet described in published literature, software must be made available to editors and reviewers. We strongly encourage code deposition in a community repository (e.g. GitHub). See the Nature Portfolio guidelines for submitting code & software for further information.

Data

Policy information about availability of data

All manuscripts must include a data availability statement. This statement should provide the following information, where applicable:

- Accession codes, unique identifiers, or web links for publicly available datasets
- A description of any restrictions on data availability
- For clinical datasets or third party data, please ensure that the statement adheres to our policy

As part of this paper all images at full resolution, all derived image data (e.g. segmentation masks), and all cell count tables will be publicly released via the NCI-recognized repository for Human Tumor Atlas Network (HTAN; https://humantumoratlas.org/) at Sage Synapse (associated Identifiers: HTAN TNP – TMA, OHSU_TMA1_004-XX) where XX represents TMA core ID. For CRC TMA, see source publication (DOI10.1016/j.cell.2022.12.028)

Human research participants

Policy information about studies involving human research participants and Sex and Gender in Research.

Reporting on sex and gender	N/A
Population characteristics	N/A
Recruitment	N/A
Ethics oversight	N/A

Note that full information on the approval of the study protocol must also be provided in the manuscript.

Field-specific reporting

Please select the one below that is the best fit for your research. If you are not sure, read the appropriate sections before making your selection.

☒ Life sciences ☐ Behavioural & social sciences ☐ Ecological, evolutionary & environmental sciences

For a reference copy of the document with all sections, see nature.com/documents/nr-reporting-summary-flat.pdf

Life sciences study design

All studies must disclose on these points even when the disclosure is negative.

Sample size	Our data consists of CyCIF images from two TMA datasets, one containing breast cancer (BC) tissue and the other containing colorectal cancer (CRC) tissue. The tissue microarrays (TMAs) CyCIF imaging data are available via HTAN (https://humantumoratlas.org/). The breast cancer (BC) TMA contains 88 cores representing 6 cancer subtypes. The colorectal cancer (CRC) TMA contains 332 cores.
Data exclusions	For the CRC TMA, any core containing a channel with a mean intensity beyond 2 standard deviations from the mean intensity of the entire TMA for that channel was dropped. This resulted in 12 cores being removed.
Replication	N/A
Randomization	Cross-validation was performed on the CRC TMA dataset to evaluate model performance on unseen data. We divide the dataset at the TMA core level by separating the cores into 5 sets of 64 TMA cores for the test sets and the remaining TMA cores are used to train 5 separate models.
Blinding	N/A

Reporting for specific materials, systems and methods

We require information from authors about some types of materials, experimental systems and methods used in many studies. Here, indicate whether each material, system or method listed is relevant to your study. If you are not sure if a list item applies to your research, read the appropriate section before selecting a response.

Materials & experimental systems

n/a	Involved in the study
☒	☐ Antibodies
☒	☐ Eukaryotic cell lines
☒	☐ Palaeontology and archaeology
☒	☐ Animals and other organisms
☒	☐ Clinical data
☒	☐ Dual use research of concern

Methods

n/a	Involved in the study
☒	☐ ChIP-seq
☒	☐ Flow cytometry
☒	☐ MRI-based neuroimaging
